# Novel clip device for prevention of bleeding after endoscopic papillectomy

**DOI:** 10.1002/deo2.51

**Published:** 2021-09-29

**Authors:** Haruo Miwa, Kazuya Sugimori, Hiromi Tsuchiya, Makoto Sugimori, Masaki Nishimura, Yuichiro Tozuka, Satoshi Komiyama, Takeshi Sato, Takashi Kaneko, Kazushi Numata, Shin Maeda

**Affiliations:** ^1^ Gastroenterological Center Yokohama City University Medical Center Kanagawa Japan; ^2^ Department of Gastroenterology Graduate School of Medicine, Yokohama City University Kanagawa Japan

**Keywords:** ampullary neoplasms, bleeding endoscopy, endoscopic papillectomy, prophylactic clipping

## Abstract

**Objectives:**

Recently, a novel clip device, SureClip^®^ (Micro‐Tech Co. Ltd., Nanjing, China), has been developed, which improved rotation and reopening performance. We aimed to assess the efficacy of the SureClip^®^ in prophylactic closure of the mucosal break after endoscopic papillectomy (EP) for ampullary neoplasm.

**Methods:**

We retrospectively reviewed the medical records of 40 patients who underwent EP for ampullary neoplasms between October 2009 and March 2020. Prophylactic closure after resection was performed using the conventional clip between 2014 and 2018, and with the SureClip^®^ after 2019. The baseline characteristics, techniques, outcomes, and complications of EP were analyzed.

**Results:**

The median age of the patients (25 males and 15 females) was 70 years. The en block resection rate was 82.5% and the curative resection rate was 80.0%. Histologically, 11 (27.5%) patients had malignancy. Prophylactic closure was performed in 29 (72.5%) patients (17 conventional clips, 12 SureClip^®^). Complications occurred in 18 (45.0%) patients, including postprocedure bleeding in 9 (22.5%) patients. However, no postprocedure bleeding was observed in the patients who received prophylactic closure using the SureClip^®^ (*p* = 0.038). All other factors were not significantly correlated with postprocedure bleeding. The duration of hospital stay after EP was significantly shorter in patients treated with the SureClip^®^ compared to those treated with a conventional clip or without clips (*p* < 0.05).

**Conclusions:**

In the present study, prophylactic clipping of the mucosal break using the SureClip^®^ was effective in preventing bleeding after EP.

## INTRODUCTION

Ampullary neoplasms, including adenomas and adenocarcinomas, show a prevalence of 0.04%–0.12% in autopsy studies,^1^ but are increasingly being diagnosed due to the continuous developments in endoscopy.^2^ Most of these lesions cause no symptoms; however, adenomas have malignant potential and should, therefore, be removed.^3^ Historically, ampullary neoplasms have been treated surgically with pancreatoduodenectomy. More recently, endoscopic papillectomy (EP) as a less invasive procedure has become the first‐line therapy for ampullary adenomas and early adenocarcinomas.^4^, ^5^, ^6^, ^7^, ^8^, ^9^

Although there are increasing reports of EP for ampullary neoplasms, studies regarding the prevention of procedure‐related complications are limited. In particular, bleeding, pancreatitis, and duodenal perforation have been reported after EP.^10,^, ^11^ Among them, postprocedure bleeding can be a serious complication that is refractory to treatment. The exposure of the mucosal break to bile or pancreatic juice is considered a factor that exacerbates the bleeding after EP. Closure of the mucosal break with endoscopic clipping devices (ECD) has been reported to be effective in the prevention of postprocedure bleeding.^12^ However, clipping with the duodenoscope is often challenging, and there is no clinical evidence as to which ECD is the best for prophylactic closure of the mucosal break after EP. A novel ECD, SureClip^®^ (Micro‐Tech Co. Ltd.), has been developed to overcome these difficulties. Its use with the duodenoscope is easier than that of existing ECDs because it possesses improved rotation and reopening performance.

The aim of this study was to clarify the efficacy of this novel clip device in the prevention of postprocedure bleeding after EP.

## METHODS

### Patients

Between October 2009 and March 2020, 41 patients underwent EP for ampullary neoplasm at the Yokohama City University Medical Center. Among them, we analyzed 40 patients for the risk of postprocedure bleeding in this retrospective study, excluding one patient who required emergency surgery due to severe intraprocedure bleeding. All EP procedures were performed after obtaining written informed consent from each patient. The study protocol was approved by the institutional review board of Yokohama City University (approval number: B200500019) and all procedures conformed to the provisions of the Declaration of Helsinki (as revised in Fortaleza, Brazil, October 2013).

### Indications for EP

All patients were diagnosed based on the results of a biopsy before EP, and all lesions suggesting an adenoma confined to the ampulla of Vater were indicated for EP. In the cases of adenocarcinoma, EP was exceptionally performed based on the patient's strong preference over other surgical procedures. Prior to EP, endoscopic ultrasound was performed to exclude an invasion of the duodenal muscularis propria or extension into the bile or main pancreatic duct. In the first years of the study period, endoscopic retrograde cholangiopancreatography (ERCP) with intraductal ultrasound was performed a few days before EP, but it was later performed simultaneously.

### Procedure of EP

All procedures were performed by three expert endoscopists specializing in ERCP (KS, TK, and HM who are supervising doctors of the Japan Gastroenterological Endoscopic Society), using the TJF‐260V (Olympus Medical Systems, Tokyo, Japan) and standard polypectomy snares. Submucosal injection was performed when the tumors showed lateral spreading. Whenever possible, the ampulla of Vater was resected en block from the hooding fold to the frenulum. A high‐frequency electrosurgical generator (ESG‐100 electrocoagulation unit; Olympus Medical Systems) in Pulse Cut Slow mode (80 W) was used for resection throughout the study period. If a remnant tumor was found, the piecemeal technique was applied using snare or hot biopsy forceps. The resected specimen was removed using alligator forceps or a catching net. A pancreatic stent was routinely placed to prevent postprocedure pancreatitis. A biliary stent was placed in the early years of the study period, whereas endoscopic sphincterotomy was adopted, as an alternative, later on. When bleeding did not stop spontaneously, endoscopic hemostasis was performed during EP.

### Prophylactic clipping

**FIGURE 1:**
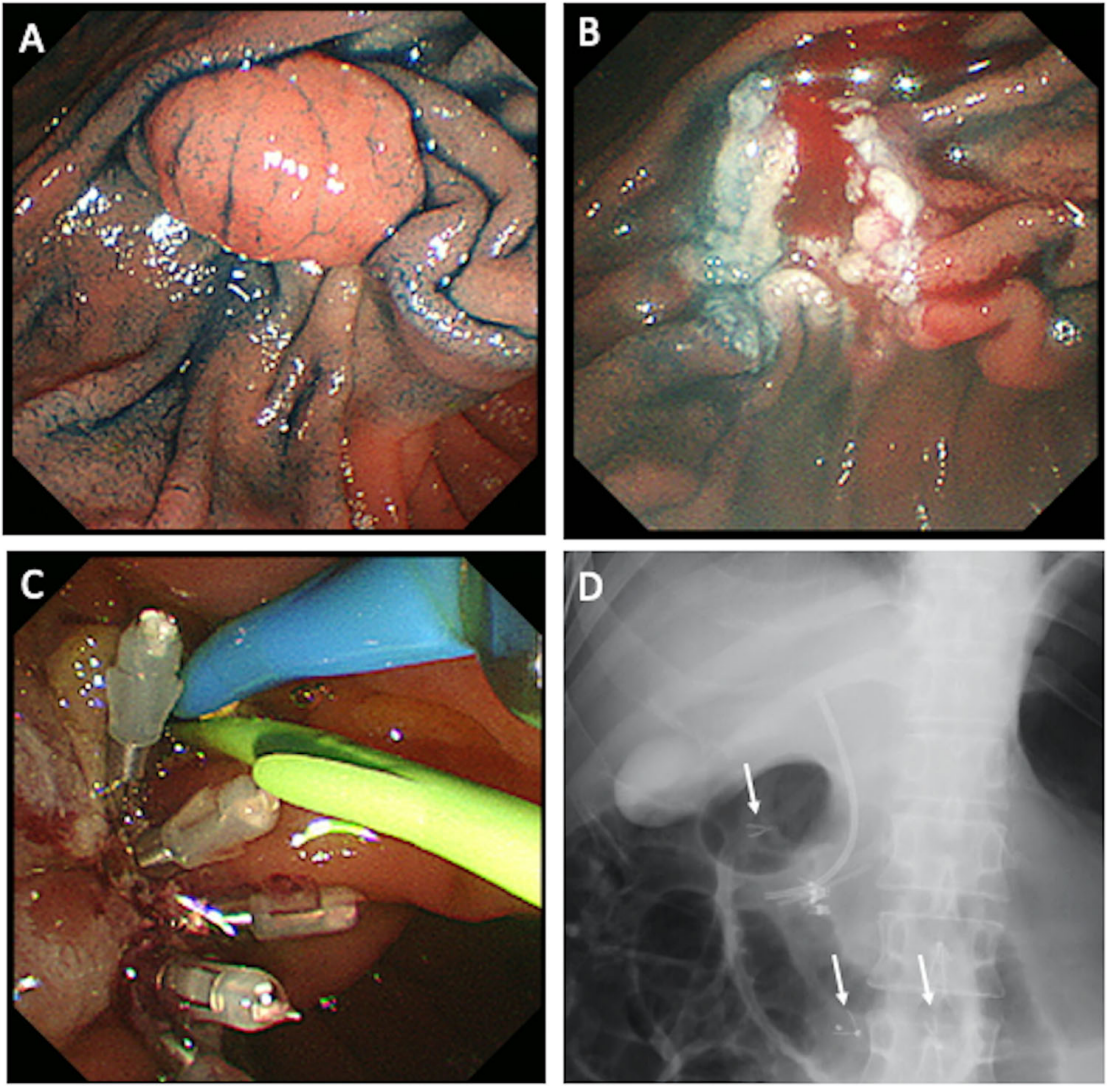
Endoscopic papillectomy of ampullary adenoma with a conventional clip. (A) A polypoid lesion at the ampulla of Vater. (B) Oozing of blood observed at the mucosal defect. (C) Prophylactic closure with conventional clip is performed. (D) Fluoroscopic image showing dropped clips in the duodenum (arrow)

**FIGURE 2:**
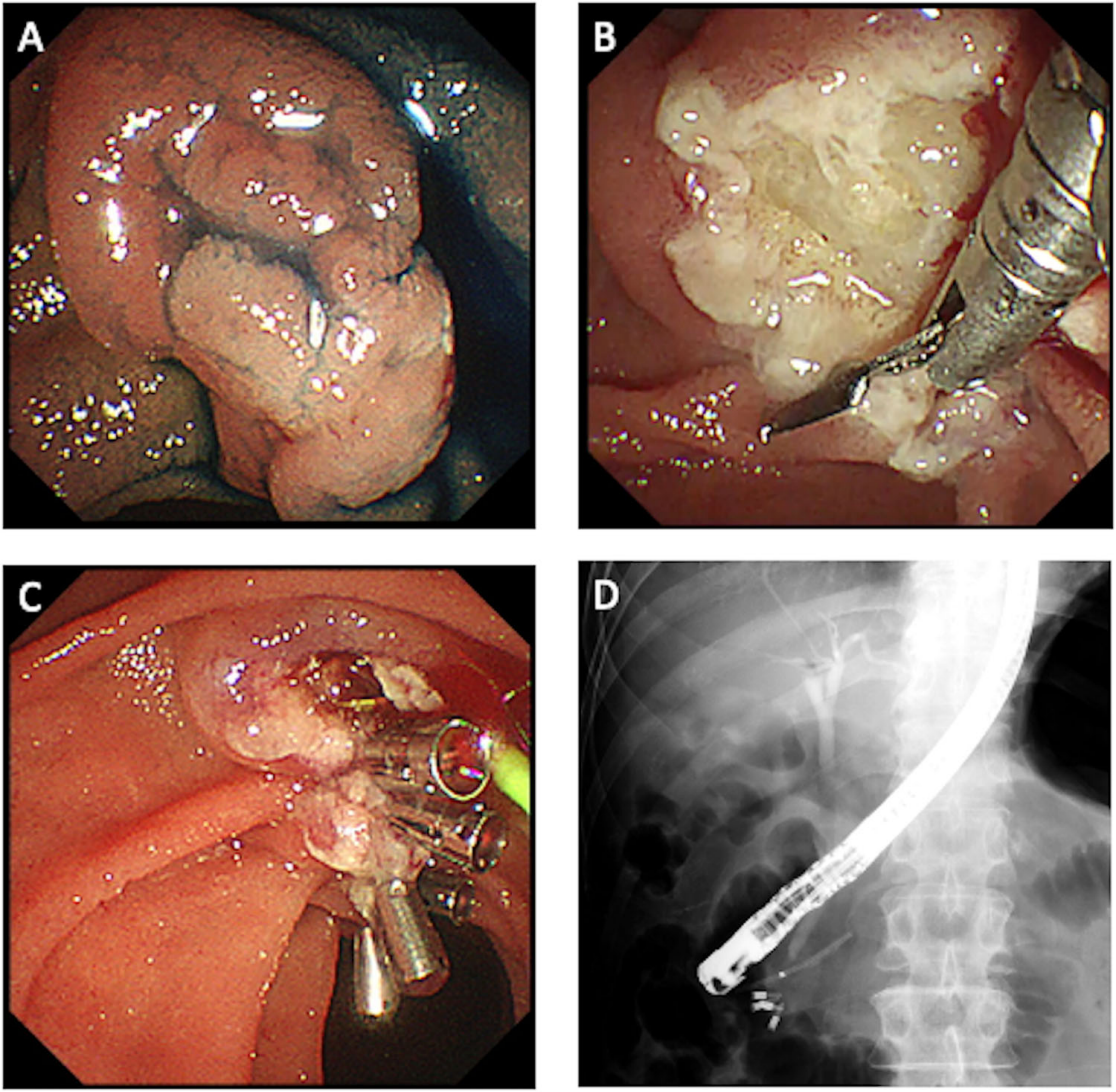
Endoscopic papillectomy for ampullary adenoma with SureClip^®^. (A) A pale and flat lesion on the anal side of the ampulla of Vater. (B) Prophylactic closure is started after *en block* resection from the anal side of the mucosal break. (C) Prophylactic closure was successfully completed. (D) Fluoroscopic image after procedure shows four clips and a pancreatic stent placed

Since mid‐2014, the clipping method using an ECD was adopted for prophylactic closure of the mucosal break and it was performed for all cases from November 2015. After resection of the papilla, clipping was first performed at the anal end to prevent expansion of the mucosal break. Next, as many clips as possible were used for clipping toward the oral side taking care to avoid overlapping of the clips and avoid closing of the orifice of the pancreatic duct by the clip at the oral end. Normal mucosa from two opposite sides was grabbed using the tips of the clips to tightly close the mucosal break. Between June 2014 and September 2018, the EZ Clip (Olympus Medical Systems) was used as a conventional clip (Figure 1); however, we used the SureClip^®^ (Micro‐Tech Co. Ltd.) since April 2019 (Figure 2; Supporting Information Video 1).

### Management after EP

After EP, all patients fasted for at least 2 days. We performed second‐look endoscopy in the early years but only carefully observed any clinical symptoms of bleeding since 2019. Oral intake was resumed in patients who did not have clinical symptoms or endoscopic findings of any complications. In uncomplicated cases, the pancreatic stent was removed one week after EP, and patients were discharged between the 8th and the 10th days.

### Complications

All complications associated with EP were evaluated according to the Cotton criteria.^13^ Postprocedure bleeding was defined as clinical evidence of melena or hematemesis after EP associated with hemoglobin decrease of 3 g/dl or more. Bleeding was classified into mild (no blood transfusion needed), moderate (up to four units of blood transfused), and severe (five units or more transfused and required angiographic or surgical intervention). When bleeding occurred, endoscopic hemostasis was initially attempted with injection of hypertonic saline–epinephrine (HSE), which consists of 3.6% sodium chloride and 0.005% epinephrine, electrical coagulation, hemostatic clipping, or polyglycolic acid sheet coating. If endoscopic hemostasis was not successful, transarterial embolization or surgery was considered. Pancreatitis was diagnosed when patients had at least two of the following findings: abdominal pain, elevated pancreatic enzyme levels, or specific findings on computed tomography. Duodenal perforation was diagnosed when there was air leakage into the abdominal or retroperitoneal cavity. Biliary tract infection, cholangitis, and cholecystitis were diagnosed according to the Tokyo Guideline 2018.^14^, ^15^

### Statistical analysis

All statistical analyses were performed using JMP pro, version 12 (SAS Institute Inc., Cary, NC, USA). Continuous variables are presented as the median with range, and categorical variables are presented as the frequency (*n*) and proportion (%). Continuous variables were compared using the Student *t*‐test and categorical variables using Fisher's exact test to identify any factors potentially associated with postprocedure bleeding. We defined a statistically significant difference as a *p*‐value <0.05.

## RESULTS

The baseline characteristics of enrolled patients are shown in Table 1. The 40 patients included 25 men and 15 women with a median age of 70 years (range 43–83). Ampullary neoplasms were classified into 34 (85.0%) polypoid lesions and 6 (15.0%) flat lesions by morphology. One patient had local recurrence of the tumor during the follow‐up period of 25 months (median, range 3–122). No metastatic lesions were recorded.

**TABLE 1:** Baseline characteristics of enrolled patients

Median age, years (range)	70 (43–83)
Sex	
Male	25 (62.5)
Female	15 (37.5)
Comorbidities	
Hypertension	7 (17.5)
Diabetes mellitus	4 (10.0)
Cardiovascular disease	3 (7.5)
Hemodialysis	1 (2.5)
Oral steroid drug	3 (7.5)
Oral antithrombotic drug	2 (5.0)
Macroscopic findings	
Elevated	34 (85.0)
Flat	6 (15.0)
Follow‐up period, month	25 (3–122)
Recurrence	1 (2.5)

*Note*: Values are *n* (%) or median (range).

The techniques and outcomes of EP are also shown in Table 2. The median procedure time was 47 min (range 17–81). En block resection was achieved in 82.5% of lesions (33/40), whereas the remaining lesions had to be resected using the piecemeal technique. The median specimen size was 21 mm (range 13–33). Curative resection, with no obvious remnant tumor in the endoscopic and histopathological examination, was achieved in 80.0% (32/40) of patients. Histological results revealed 11 (27.5%) malignancies (5 [12.5 %] adenocarcinomas within adenomas and 6 [15.0%] adenocarcinomas), 27 (67.5%) adenomas, and 2 (5.0%) hyperplasias.

**TABLE 2:** Techniques and outcomes of endoscopic papillectomy

Procedure time, min (range)	47 (17–81)
Simultaneous ERCP	20 (50.0)
En block resection	33 (82.5)
Curative resection	32 (80.0)
Specimen size, mm	21 (13–33)
Histology	
Adenoma	27 (67.5)
Adenocarcinoma within adenoma	5 (12.5)
Adenocarcinoma	6 (15.0)
Hyperplasia	2 (5.0)
Sphincterotomy for bile duct	15 (37.5)
Biliary stenting	22 (55.0)
Pancreatic stenting	36 (90.0)
Prophylactic clipping	29 (72.5)
Conventional clip	17 (42.5)
SureClip^®^	12 (30.0)
Number of clips, median, range (range)	5 (2–9)
Hemostasis during procedure	5 (12.5)
Complications associated with EP	18 (45.0)
Postprocedure bleeding	9 (22.5)
Mild/moderate/severe	7 / 0 / 2
Pancreatitis	6 (15.0)
Duodenal perforation	3 (7.5)
Biliary tract infection	3 (7.5)
Hemostasis for postprocedure bleeding	
Hypertonic saline–epinephrine injection	6 (15.0)
Coagulation	6 (15.0)
Clipping	3 (7.5)
Polyglycolic acid seat	1 (2.5)
Transarterial embolization	1 (2.5)
Surgery	0 (0)
Blood transfusion for postprocedure bleeding	2 (5.0)
Mortality	0 (0)
Duration of fasting time after EP, day (range)	3 (2–14)
Duration of hospital stay after EP, day (range)	10 (7–25)

*Note*: Values are *n* (%) or median (range).

Abbreviations: EP, endoscopic papillectomy; ERCP, endoscopic retrograde cholangiopancreatography.

Prophylactic clipping was performed in 29 (72.5%) patients. Conventional clip and SureClip^®^ were used in 17 (42.5%) and 12 (30.0%) patients, respectively. Among the patients not undergoing clipping, three received prophylactic polyglycolic acid sheet coating, whereas nine patients had no prophylactic procedure.

During EP, five (12.5%) patients received endoscopic hemostasis for intraprocedure bleeding. Among them, complete hemostasis was achieved by endoscopic modalities using HSE injection, hemostatic clipping, and bipolar hemostatic forceps (Hemostat‐Y; Pentax, Tokyo, Japan). Postprocedure bleeding occurred in nine (22.5%) patients; within 2 days of EP in eight patients, and on the seventh day in one patient. Mild and severe bleeding occurred in seven and two patients, respectively, as per the Cotton criteria. Eight patients were treated only by endoscopic hemostasis, and one required transarterial embolization. As for other complications, pancreatitis was observed in six patients (15%), duodenal perforation in three patients (7.5%), and biliary tract infection in another three patients (7.5%). These complications were not significantly correlated with the presence or absence of prophylactic clipping or the type of clip. There was no mortality associated with the procedure.

Table 3 shows the results of a univariate analysis comparing the characteristics of patients with and without postprocedure bleeding. In terms of prophylactic procedure, four patients with postprocedure bleeding were treated with conventional clips, and five did not receive clipping. In contrast, patients who underwent prophylactic clipping using SureClip^®^ showed no postprocedure bleeding (*p* = 0.038). All other factors were not significantly correlated with postprocedure bleeding.

**TABLE 3:** Univariate analysis of clinicopathological factors for with/without postprocedure bleeding

	Postoperative bleeding (−)*N* = 31	Postoperative bleeding (+)*N* = 9	*p*‐Value^a^
Age (≥75 yr/<75 yr)	11 (35.5)/20 (64.5)	1 (11.1)/8 (88.9)	0.233
Sex (male/female)	18 (58.1)/13 (41.9)	7 (77.8)/2 (22.2)	0.440
Comorbidities			
Hypertension	5 (16.1)	2 (22.2)	0.645
Diabetes mellitus	3 (9.7)	1 (11.1)	1.000
Cardiovascular disease	3 (9.7)	0 (0)	1.000
Hemodialysis	1 (3.2)	0 (0)	1.000
Oral steroid drug	2 (6.5)	1 (11.1)	0.545
Oral antithrombotic drug	1 (3.2)	1 (11.1)	0.404
Specimen size (≥20 mm)	18 (58.1)	5 (55.6)	1.000
Elevated‐shaped	25 (80.6)	9 (100)	0.303
Malignancy	8 (25.8)	3 (22.6)	0.686
Simultaneous ERCP	16 (51.6)	4 (44.4)	1.000
En block resection	24 (77.4)	9 (100)	0.175
Procedure time (≥60 min)	8 (25.8)	1 (11.1)	0.654
Sphincterotomy for bile duct	14 (45.2)	1 (11.1)	0.117
Biliary stenting	15 (48.4)	7 (77.8)	0.149
Pancreatic stenting	27 (87.1)	9 (100)	0.557
Prophylactic clipping			
Conventional clip	12 (38.7)	5 (55.6)	0.456
SureClip^®^	12 (38.7)	0 (0)	0.038
No clips	7 (22.6)	4 (44.4)	0.227
Number of clips (≥5 clips)	16 (51.6)	3 (33.3)	0.457
Hemostasis during procedure	3 (9.7)	2 (22.2)	0.311

*Note*: Values are *n* (%).

Abbreviation: ERCP, endoscopic retrograde cholangiopancreatography.

^a^ Fisher's exact test.

## DISCUSSION

This study showed that prophylactic clipping with SureClip^®^ is an effective method for preventing postprocedure bleeding. The overall rate of postprocedure bleeding was 22.5% (9/40) in patients after EP; however, none of the patients treated with the SureClip^®^ had statistically significant bleeding (*p* = 0.038).

EP was first reported in 1993 as a safe and effective treatment for ampullary adenoma.^4^ Recently, the indications for EP have been extended to patients with adenocarcinoma confined to the ampulla of Vater.^16^ EP is less invasive than surgery; however, due to the anatomical characteristics of the ampulla region, it has a higher risk of complications than endoscopic resection of gastric or colorectal neoplasms.^17,^, ^18^ In particular, bleeding is a relatively frequent complication after EP that has been reported in 17%–32% of patients, even when limited to studies in recent years.^19^, ^20^, ^21^, ^22^ There are several explanations for the high incidence of bleeding after EP. First, the papilla of Vater receives substantial blood supply from several arteries.^23^ Second, the mucosal break after EP is exposed to pancreatic juice and bile from the orifice of the ducts. These digestive juices can delay mucosal break healing by interfering with epithelialization.

**FIGURE 3:**
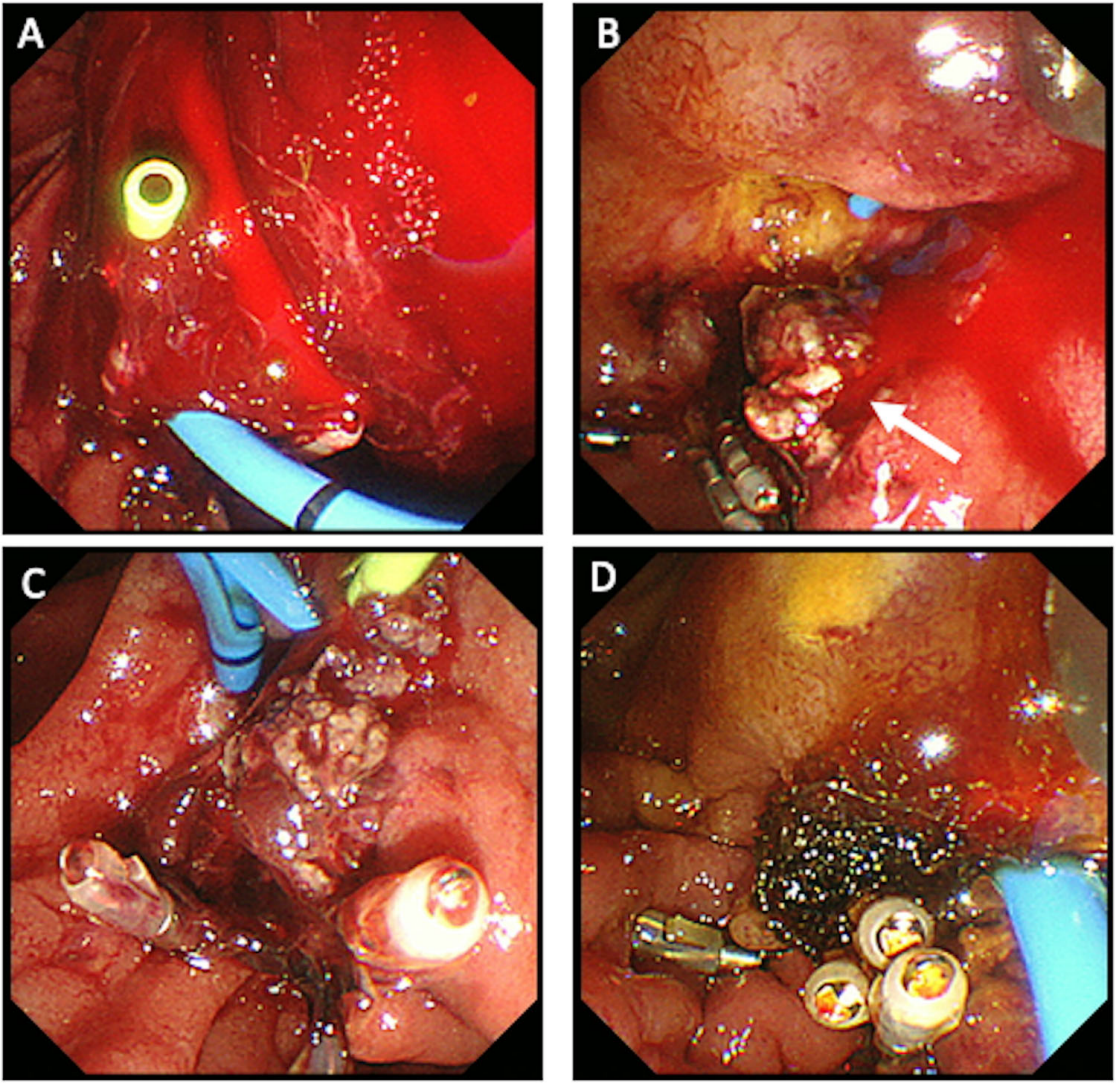
Emergency endoscopy on the next day after papillectomy (same patients as in Figure 1). (A) Endoscopic image shows large amount of hematoma and active bleeding in the second part of duodenum. (B) The mucosal defect is opened, and pulsatile bleeding is observed from the exposed vessel (arrow). (C) Hemostasis using bipolar forceps is successfully performed. (D) Polyglycolic acid sheet is packed in the mucosal break after hemostasis

When bleeding occurred after EP, hemostasis was performed endoscopically. The methods were chosen according to the degree of bleeding. For mild oozing alone, we sprayed saline solution containing epinephrine. When persistent oozing or arterial bleeding occurred near the margin of the mucosal defect, HSE was injected into the adjacent normal tissue. Electrical hemostatic forceps were used when an arterial bleeding point or injured vessel could be directly observed. Bipolar forceps were used to reduce the depth of burning and tissue destruction.^24,^, ^25^ However, bleeding from the mucosal break after EP is often refractory (Figure 3). In one case where the endoscopic hemostasis was not successful, transarterial embolization was performed by a radiologist as an emergency intervention.

As for preventing postprocedure bleeding after colorectal endoscopic mucosal resection, prophylactic clipping has been reported as an effective procedure.^26^, ^27^, ^28^ Ayoub *et al*. previously reported the efficacy of this procedure in their meta‐analysis,^27^ and Osada *et al*. has described accelerated healing of mucosal defect using clipping closure.^26^ However, only a few methods have been reported for the prevention of bleeding after EP. Kagawa *et al*. reported the efficacy of prophylactic clipping in a prospective pilot study.^12^ In that report, only 5% (2/40) of patients with clipping experienced postprocedure bleeding, while 22.5% of patients without clipping experienced bleeding. However, they also reported that the clipping closure technique after EP is relatively challenging.

We used EZ Clip as a conventional clip system between 2012 and 2018. This clip is widely used in Japan for endoscopic hemostasis with forward‐viewing endoscopes because it reduces the cost by using a reloadable system. However, it is difficult to apply the EZ Clip with a duodenoscope for several reasons. First, we cannot use the elevator of the duodenoscope while opening and rotating the clip. Therefore, it is necessary to perform fluoroscopy to avoid injuring the duodenal wall with the tips of the clip. Second, it is impossible to reopen the clip after having grabbed the tissue. If the clip has been deployed in an inappropriate place, it is difficult to reposition it in the correct location afterward. Therefore, the clips must be deployed accurately on the first attempt. To overcome these problems, we adopted the SureClip^®^ for prophylactic clipping after EP from April 2019. This ECD has been developed with the following advantages: it rotates smoothly, is reopenable after grabbing the tissue, and is disposable. With the SureClip^®^, we can rotate the clip to adjust for the direction of the mucosal break while observing endoscopically by lifting the elevator. The reopenable system enables us to release the clip after grasping normal tissue adjacent to the mucosal defect. Moreover, the disposable system reduces the time required to change the ECD during the procedure.

There are two possible explanations for the absence of postprocedure bleeding when using SureClip^®^ in our study. First, this sophisticated ECD, with its excellent ability to rotate and reopen the clip, enables the endoscopist to place each clip in the most appropriate position by grasping normal tissue adjacent to the mucosal break. With the conventional clip, postprocedure bleeding occurred from a reopened mucosal break after spontaneous removal of the clips. Second, reducing the procedure time for prophylactic clipping may prevent postprocedure bleeding. The mucosal break caused by EP rapidly widens, and this makes clipping closure more difficult as more time elapses. The advantages of SureClip^®^, such as reopenability and rotability, reduce the time required for prophylactic clipping and help in effective closure of mucosal break.

Regarding the disadvantage of prophylactic clipping after EP, it may result in accidental obstruction of the orifice. We routinely place a pancreatic stent to identify the orifice of the main pancreatic duct before placing the last clip to prevent pancreatitis. Among the patients in whom we could not place a pancreatic stent, one patient who received prophylactic closure with the SureClip^®^ experienced severe pancreatitis after EP. Although, no significant correlation was observed between prophylactic closure with the SureClip^®^ and complications other than bleeding in the present study, tight closing with the SureClip^®^ might have promoted mucosal break healing that resulted in obstruction of the orifice. Therefore, prophylactic clipping is recommended to be performed by maintaining a sufficient distance from the orifice of the pancreatic duct in cases which a pancreatic stent cannot be placed.

This study has several limitations. First, it was retrospective, and the number of patients was limited. Despite the retrospective design, selection bias is expected to have been minimal because the prophylactic procedure was not selected on a case‐by‐case basis but was conducted using consecutive patients. Multivariate analysis was not performed owing to the low number of cases. Second, the incidence of postprocedure bleeding in our patients without prophylactic clipping or a conventional clip was higher than that reported in previous studies. The frequent use of biliary stenting may have influenced the occurrence of postprocedure bleeding in these patients.

In conclusion, our present study demonstrated that the prophylactic closure with SureClip^®^ was effective in preventing postprocedure bleeding following EP. We believe that the improvement in ECD makes EP safer and easier, further supporting it as a minimally invasive treatment for ampullary neoplasms.

## CONFLICT OF INTEREST

The authors declare that there is no conflict of interest.

## ETHICS STATEMENT

Written informed consent based on the Helsinki Declaration (1964, 1975, amended in 1983, 2003, and 2008) was obtained from all patients. The Review Board of Yokohama City University reviewed and approved the study protocol.

## FUNDING INFORMATION

None.

## Supporting information


**Supporting Video 1**. The video shows endoscopic papillectomy for ampullary adenoma. Prophylactic closure with SureClip^®^ is performed after *en block* resection. At the end of the procedure, endoscopic sphincterotomy is performed, and the pancreatic stent is placed.
